# CD73/NT5E is a target of miR-30a-5p and plays an important role in the pathogenesis of non-small cell lung cancer

**DOI:** 10.1186/s12943-017-0591-1

**Published:** 2017-02-03

**Authors:** Jianjie Zhu, Yuanyuan Zeng, Wei Li, Hualong Qin, Zhe Lei, Dan Shen, Dongmei Gu, Jian-an Huang, Zeyi Liu

**Affiliations:** 1 Department of Respiratory Medicine, the First Affiliated Hospital of Soochow University, Suzhou, 215006 People’s Republic of China; 2 Institute of Respiratory Diseases, Soochow University, Suzhou, 215006 China; 3 Department of Oncology, the First Affiliated Hospital of Soochow University, Suzhou, 215006 China; 4 Department of Cardiothoracic Surgery, the First Affiliated Hospital of Soochow University, Suzhou, 215006 China; 5 Laboratory of Cancer Molecular Genetics, Medical College of Soochow University, Suzhou, 215123 China; 6 Department of Pathology, the First Affiliated Hospital of Soochow University, Suzhou, 215006 China

**Keywords:** Non-small cell lung cancer, miRNA, CD73, miR-30a-5p, EGFR

## Abstract

**Background:**

CD73 (ecto-5′-nucleotidase) is implicated in the development of many types of cancer. CD73 inhibitors are currently being tested in clinical trials for the treatment of cancer. Understanding the molecular and cellular actions of CD73 inhibitors is the key to improving this line of therapy.

**Methods:**

Quantitative real-time PCR (qRT-PCR) was used to detect the expression of *CD73* and miR-30a-5p; Western blot and immunohistochemical assays were used to investigate the levels of CD73 and other proteins. Flow cytometry was used to determine cell cycle stage and apoptosis. CCK-8 and clonogenic assays were used to investigate cell proliferation. Wound healing, migration and invasion assays were used to investigate the motility of cells. A lung carcinoma xenograft mouse model was used to investigate the in vivo effects of CD73 and miR-30a-5p.

**Results:**

In the present study, we found that CD73 is overexpressed and miR-30a-5p is underexpressed in non-small cell lung cancer tissues compared with adjacent noncancerous. Further, we showed that CD73 is a direct target of miR-30a-5p by luciferase reporter assays, qRT-PCR and western blot analysis. We also found that overexpression of miR-30a-5p in these non-small cell lung cancer cell lines inhibited cell proliferation in vitro and in vivo. Moreover, the epithelial-to-mesenchymal phenotype was suppressed and cell migration and invasion were inhibited; these effects were brought about via the EGF signaling pathway.

**Conclusions:**

Our findings reveal a new post-transcriptional mechanism of CD73 regulation via miR-30a-5p and EGFR-related drug resistance in non-small cell lung cancer.

**Electronic supplementary material:**

The online version of this article (doi:10.1186/s12943-017-0591-1) contains supplementary material, which is available to authorized users.

## Background

Lung cancer is the leading cause of cancer-related deaths in China and other countries [1, 2]. Non-small cell lung cancer (NSCLC) accounts for 85% of lung cancers. Despite the advances in cancer research and treatment, the prognosis of NSCLC is still poor, with the 5-year survival rate being only 15% [3]. Although new drugs such as gefitinib and erlotinib have been shown to be beneficial, especially in patients with sensitive target mutations, the survival and outcome have not changed dramatically. Therefore, it is important to understand the pathogenesis of NSCLC and identify new treatment targets.

Recently, the purinergic signaling pathway, in which extracellular ATP, ADP and adenosine are the main signaling molecules, has emerged as an important player in cancer progression [4]. Purinergic signaling is a multistep coordinated cascade that involves stimulated release of ATP/ADP, triggering of signaling events via P2 receptors, and inactivation of the nucleotide to form adenosine, which binds to its own activated P1 receptors and influences cell survival, proliferation and cell motility [5]. Ecto-5′-nucleotidase (CD73), a component of the purinergic signaling pathway, is a 70-kD glycosylphosphatidylinositol-anchored cell surface protein encoded by the *NT5E* gene that plays a crucial role in switching on adenosinergic signaling. CD73 has both enzymatic and non-enzymatic functions in cells [6]: as a nucleotidase, CD73 catalyzes the hydrolysis of AMP into adenosine and phosphate, and CD73-generated adenosine plays an important role in tumor immunoescape [7]; moreover, CD73 also functions as a signal and adhesive molecule that can regulate cell interaction with extracellular matrix components, such as laminin and fibronectin, to mediate the invasive and metastatic properties of cancers [8, 9]. Both the enzymatic and non-enzymatic functions of CD73 are involved in cancer-associated processes and are not completely independent of each other [10]. There is ample evidence to show that CD73 is a key regulatory molecule in cancer development and is overexpressed in many cancers, including leukemia, glioblastoma, melanoma, ovarian cancer, esophageal cancer, prostate cancer and breast cancer [10]. CD73 expression is also associated with certain clinical characteristics and the prognosis of cancer patients [9, 11–15]. In particular, due to its favorable effects in tumor-bearing mouse models, which have not been investigated in the clinic, anti-CD73 therapy is now a promising approach for cancer treatment in the future [16, 17]. However, the role of CD73 in lung cancer remains unclear. Moreover, despite its functional importance, little is known about the transcriptional regulation of CD73 [18–21].

Studies have shown that the prognosis of cancer is closely related to the altered expression of miRNAs in cancer tissues and specific expression signatures or panels [22], which can also be used to classify human cancers [23] and distinguish between tumor subtypes [24]. Recent research has shown that alteration in miRNA expression may be involved in the regulation of epithelial-to-mesenchymal transition in tumor progression [25]. In particular, there is some evidence that miRNAs are closely related to the development of human lung cancer [26, 27]. In our recent study, we used miRNA arrays to demonstrate the impact of significant miRNAs on cellular pathways and biological processes, and showed that miR-30a-5p expression was significantly downregulated in NSCLC tissues [28]. To identify more novel targets of miR-30a-5p that may play a role in NSCLC, in the present study, we predicted its target mRNAs using computational algorithms. Interestingly, miR-30a-5p was one of only two miRNAs that could bind to the 3′-UTR of CD73 mRNA. Thus, miR-30a-5p may be involved in the regulation of CD73 in cancer progression.

Here, we aimed to evaluate the role of CD73 in the tumorigenesis of NSCLC, and to explore the possible role of miR-30a-5p in CD73 dysregulation in lung carcinogenesis.

## Results

### CD73 is frequently overexpressed in NSCLC tissues and cell lines

**Fig. 1:**
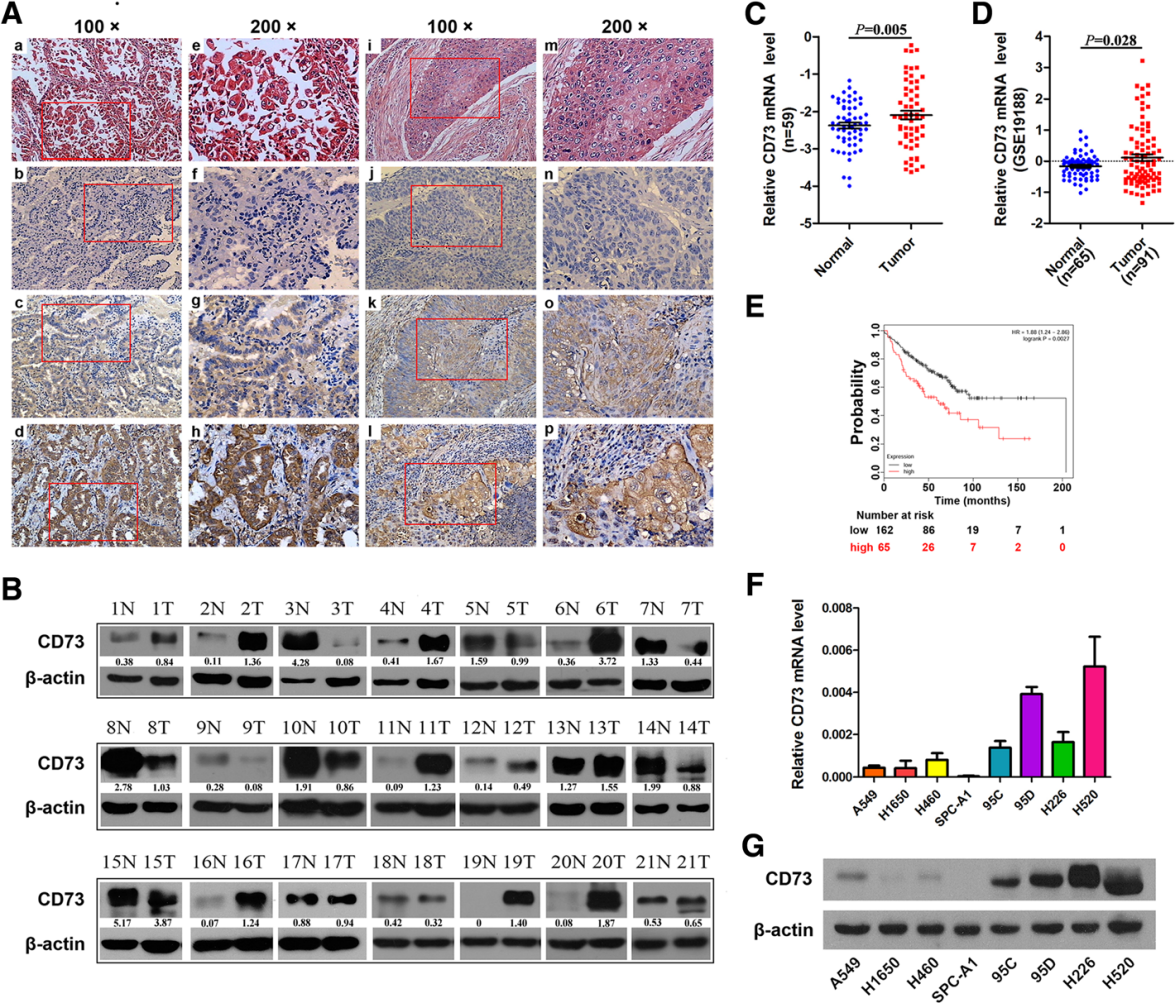
CD73 is frequently overexpressed in NSCLC. **a** Twenty-four formalin-fixed and paraffin-embedded NSCLC tissues were subjected to H & E staining and IHC analyses of the CD73 protein. Representative images are shown of H & E staining (*a* & *e*, *i* & *m*), the corresponding CD73 antibody staining (*b*–*d*, *f*-*h* and *j*–*l*, *n*-*p*) in adenocarcinomas and squamous cell carcinomas, respectively. **b** Western blot analysis of the CD73 protein levels in 21 randomly selected NSCLC tissues and paired noncancerous lung tissues; β-actin was used as an internal control. **c** CD73 mRNA levels in 59 NSCLC tissues and paired noncancerous lung tissues. **d** Relative CD73 mRNA expression levels in NSCLC tumors and adjacent normal lung tissues in a public data set (GSE19188). **e** Effect of the CD73 expression level on overall survival in 227 lung cancer patients who did not undergo chemotherapy or radiotherapy was analyzed, and Kaplan-Meier plots were generated using a Kaplan-Meier Plotter (http://www.kmplot.com). **f** and **g** The level of CD73 in human NSCLC cells was detected by qRT-PCR and Western blot, respectively. Abbreviations: N, paired noncancerous lung tissues, T, non-small cell lung cancer tissues. ^*^ *P* < 0.05

The first goal of this work was to examine the expression of CD73 protein levels in 24 NSCLC, including 12 adenocarcinoma and 12 squamous cell carcinoma, by IHC. We found that CD73 is largely located in the cell membrane and cytoplasm of NSCLC cells (Fig. 1a); levels of CD73 were high in 15 cases (14/24 = 58.33%). Further, we analyzed CD73 expression in lysates from 21 freshly harvested tissue samples of NSCLC patients by western blotting compared with matched noncancerous tissues. Among 21 randomly selected NSCLC and paired noncancerous lung tissues, 12 tumors (57.14%) showed an increase in CD73 protein (Fig. 1b). Moreover, we detected CD73 mRNA expression in 59 paired NSCLC tissues and adjacent noncancerous lung tissues: the CD73 mRNA levels were significantly higher in NSCLC tissues than adjacent noncancerous lung tissues (Fig. 1c). Additionally, a public data set (Gene Expression Omnibus, GSE19188) containing 91 NSCLC tissues and 65 normal lung tissues also showed that CD73 mRNA expression was upregulated in human NSCLC tissues (Fig. 1d). No significant difference in the CD73 mRNA level was observed between NSCLCs when they were classified according to age, gender, smoking habit of the patient, TNM stage and lymph node invasion; however, significant differences were observed according to the histological characteristics (Additional file 1: Table S1). We used the Kaplan-Meier Plotter online database [29] to determine the effects of CD73 expression in lung cancer patients and plotted a Kaplan-Meier survival curve of NSCLC patients with low or high expression of CD73. In 227 cases in which neither chemotherapy nor radiotherapy were performed, high expression of CD73 was associated with lower survival rates (Fig. 1e). We also detected CD73 mRNA and protein expression in 10 NSCLC cell lines by qRT-PCR and western blot analysis (Fig. 1f and g). Thus, CD73 expression was frequently higher in NSCLC tissues and cell lines than in the normal cell samples.

### Knockdown of CD73 inhibits in vitro cell growth, cell cycle progression and migration of NSCLC cells

**Fig. 2:**
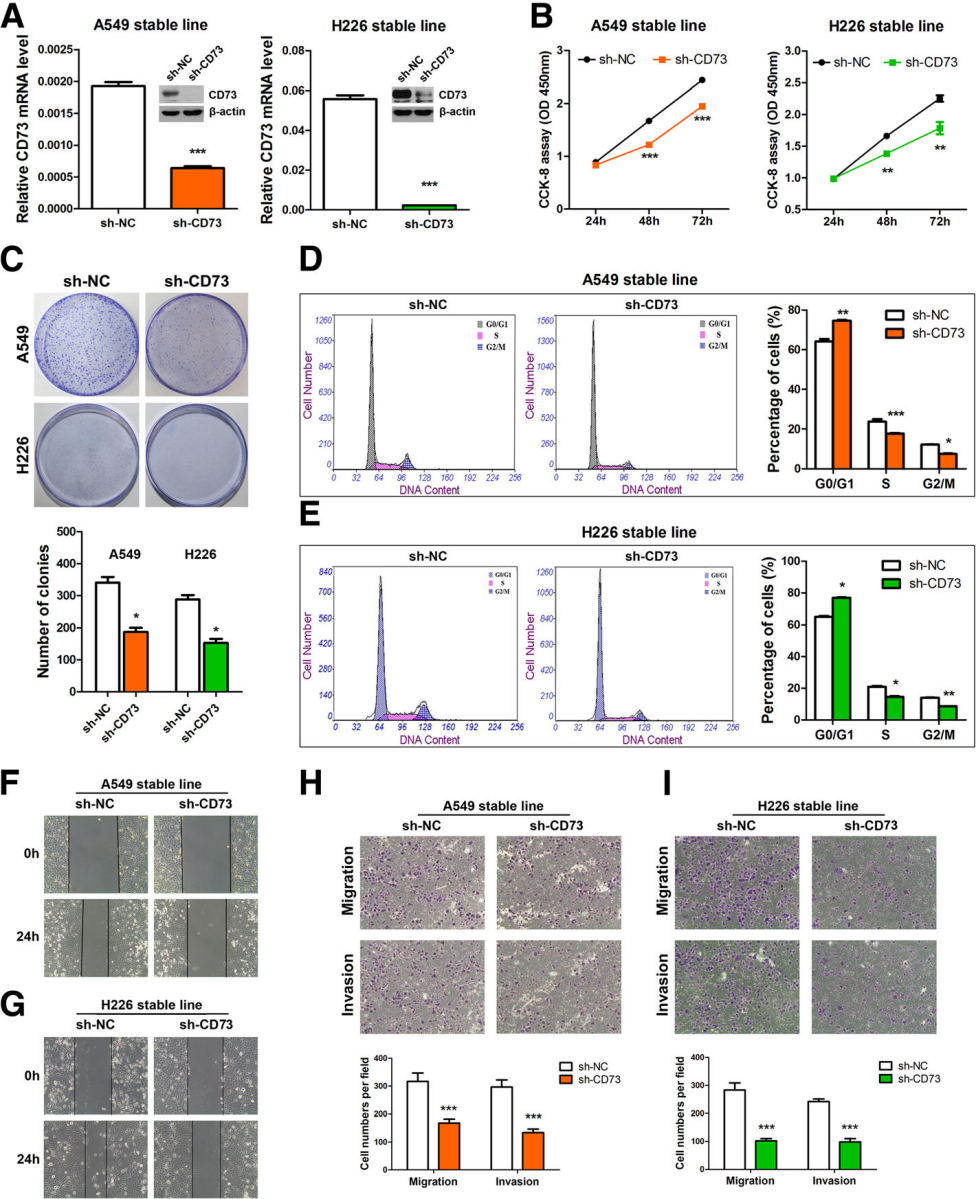
Silencing of CD73 inhibits NSCLC cell proliferation and motility. **a** CD73 mRNA and protein levels in stable A549 and H226 cells. **b** CCK-8 assay of cell viability in NSCLC cell lines; cell viability was determined at 24, 48 and 72 h. **c** Representative images of clonogenic analysis of cell proliferation in NSCLC cells. *Bar charts* showing clonogenic growth of cells. **d** and **e** Flow cytometric analysis of NSCLC cell lines (CD73-silenced cells vs. NC cells). Cells were harvested at 72 h after transfection and stained with propidium iodide. The percentage of cells in each cell cycle phase is shown in the inset of each panel, in which the values represent the mean ± SD of three measurements. **f** and **g** Wound healing assay was performed to observe the role of CD73-silenced cells, the speed with which cells migrated towards the scratch was lower in sh-CD73-transfected cells than in control cells. **h** and **i** CD73 silencing inhibits invasion and migration of NSCLC cells. CD73-silenced NSCLC cells were allowed to migrate through an 8-μM pore Transwell. The cells that migrated were stained and counted in at least three microscopic fields (magnification, ×100). Then, the cells were treated as above and allowed to invade through the Matrigel-coated membrane in Transwells. Invasive cells were stained and counted under a light microscope. ^*^ *P* < 0.05; ^**^ *P* < 0.01; ^***^ *P* < 0.001

The expression level of CD73 mRNA and protein was significantly reduced after the stable transfection of A549 and H226 cells with two CD73 short hairpin RNAs (shRNAs) (Fig. 2a, Additional file 2: Figure S1). The CCK-8 assay showed that cell growth was significantly inhibited in cells with stable knockdown of CD73 compared with the control cells at 48 h and 72 h after transfection (Fig. 2b). We confirmed these findings by performing a clonogenic assay (Fig. 2c). Moreover, flow cytometry results indicated that the proportion of cells in the G0/G1 phase was significantly higher and the proportion of cells in the S phase was significantly lower in CD73-silenced cells than in the control cells (*P* < 0.05, Fig. 2d and e). These results indicate that CD73 inhibits cell proliferation in NSCLC cells via its effects on the cell cycle.

The wound healing assay was performed to observe the effects of sh-CD73 transfection in NSCLC cells. The sh-CD73-transfected cells migrated towards the scratch more slowly than did the control cells (Fig. 2f and g). Transwell assay of the NSCLC stable cells lines further indicated that loss of CD73 considerably suppressed the migratory ability of NSCLC cells (Fig. 2h and i). Thus, CD73 may have oncogenic roles in NSCLC.

### CD73 and its associated pathways may be useful as therapeutic targets

The specific tyrosine kinase targets of CD73 was screened using a human RTK phosphorylation array on CD73 silenced and control A549 cells. The results showed that phosphorylation of the EGFR family was downregulated in CD73 silenced cells compared to control. (Additional file 3: Figure S2 and Additional file 4: Table S2). Western blots confirmed that phosphorylation of EGFR was indeed significantly altered in the A549 and H226 cell lines.

**Fig. 3:**
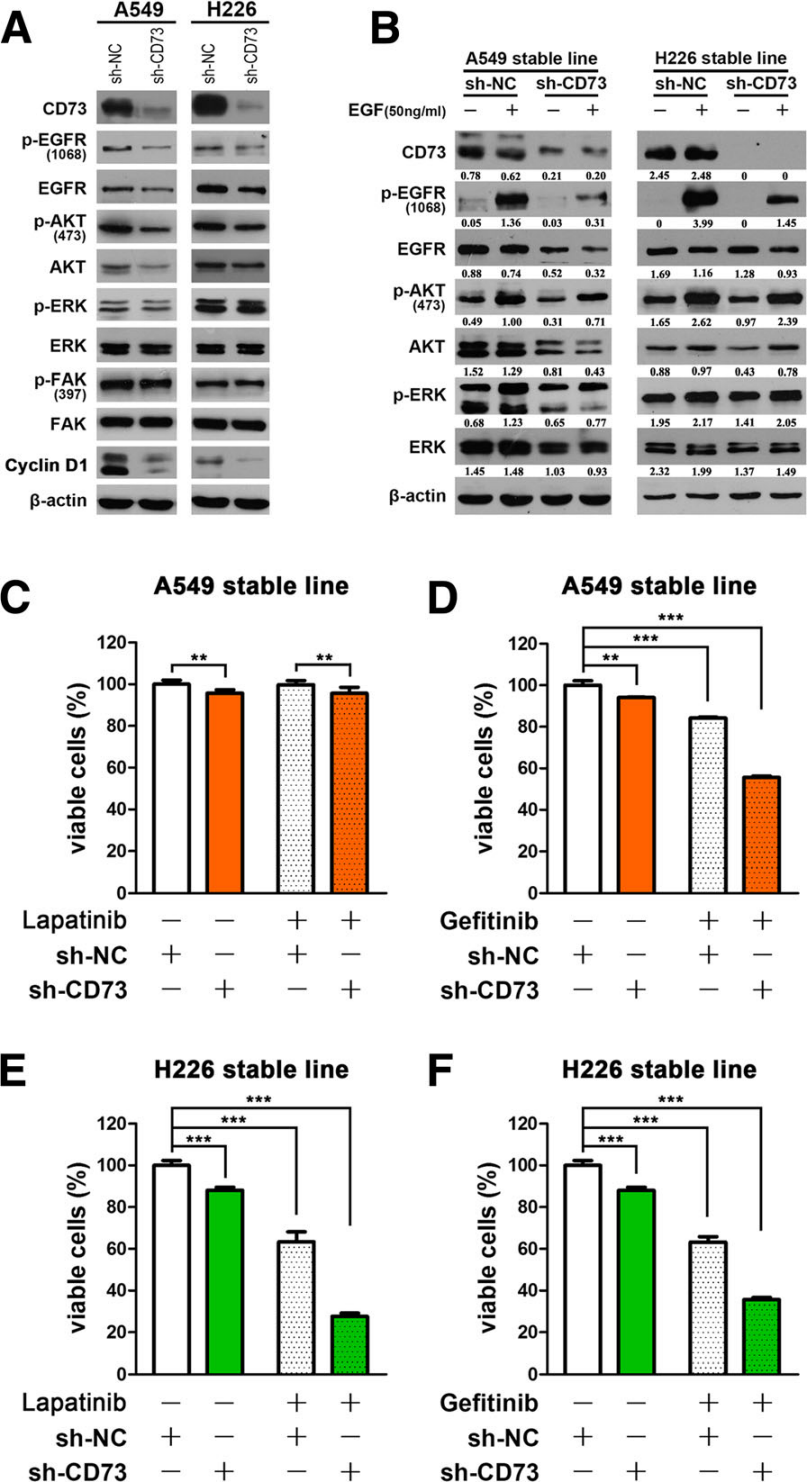
CD73 knockdown increased the sensitivity of NSCLC cells to gefitinib and lapatinib. **a** The expression of proteins in the CD73 and EGFR signaling pathways was detected in the stable A549 and H226 cell lines. Our data showed that p-EGFR and p-AKT expression is significantly decreased in CD73-silenced cell lines compared with the control cells. **b** After serum starvation for 24 h, CD73-silenced A549 and H226 cells were treated with or without EGF (50 ng/ml) for 30 min. The expression levels of CD73, p-EGFR, EGFR, p-AKT, AKT, p-ERK and ERK were analyzed by western blot analysis. **c**–**f** CD73 knockdown increased the sensitivity of NSCLC cells to gefitinib and lapatinib. The A549 and H226 cell lines were transfected with 10 μM gefitinib and 20 μM lapatinib for 48 h, respectively. After the aforementioned treatments, cell viability was assessed using CCK-8 assays. The data shown represent the mean ± SD values of four replicate experiments. All the data were obtained from three independent experiments and are shown as the mean ± SD values. ^**^ *P* 0.01; ^***^ *P* < 0.001

Overexpression of CD73 is associated with resistance to antitumor agents [30, 31]. Additionally, recent studies have shown that CD73 also affects tumor cells via the EGFR signaling pathway in breast cancer and colorectal cancer [11, 32]. EGFR is expressed more abundantly in malignant than in normal tissue, and in the case of NSCLC, EGFR expression is higher than that in normal tissue in 40–80% of the cases [33]. This makes EGFR an important target for lung cancer therapy. Based on the literature, we hypothesized that dysregulation of CD73 via the EGFR signaling pathway and knockdown of CD73 expression can sensitize NSCLC cells to therapeutic agents. First, we detected the molecular expression of EGFR and downstream signaling molecules. As illustrated in Fig. 3a, our data showed that EGFR, p-EGFR and p-AKT expression is significantly decreased in the CD73-silenced cells than in the control cells. Further, it was shown that in the stable cell lines with CD73 knockdown, the EGF-induced increase in the p-EGFR level was inhibited (Fig. 3b).

To explore the effects of knockdown of CD73 on cell growth, cell cycle progression and migration, and to determine if these events were mediated by adenosine, we used the CD73 inhibitor APCP and the adenosine analogue NECA. The results showed that treatment with APCP had a similar effect as sh-CD73 suppression in A549 cells, but had no effect in H226 cells (Additional file 5: Figure S3). Similarly, treatment with exogenous NECA increased EGFR signaling in NSCLC cell lines. These results implied that non-enzymatic functions of CD73 are important in H226 cells.

In the A549 stable cell line, the viability of the cells decreased by 50% when they were exposed to gefitinib (*P* < 0.001, Fig. 3d). Similar synergistic effects were not observed when the cells were exposed to lapatinib compared with gefitinib (Fig. 3c). In the H226 stable cell line, the viability of cells was reduced by approximately 30% in response to gefitinib and lapatinib (Fig. 3e and f); significant changes were not observed in control NSCLC cells exposed to gefitinib or lapatinb (Additional file 6: Figure S4). Our data seem to indicate that CD73 inhibition could improve the clinical effects of EGFR-TKI, especially in squamous cell lung carcinoma.

### miR-30a-5p expression is downregulated in NSCLC tissues and cell lines

**Fig. 4:**
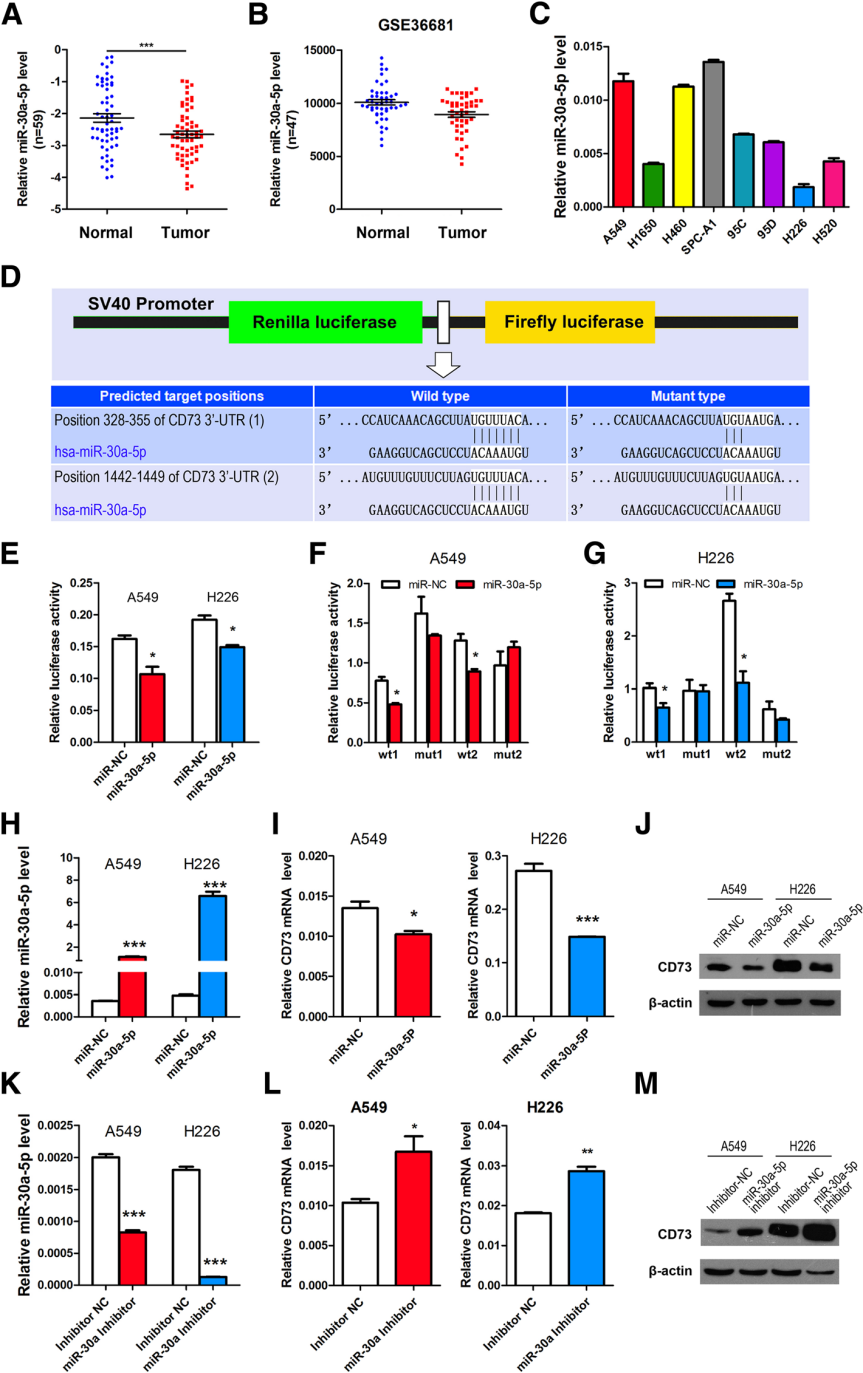
miR-30a-5p expression is decreased in NSCLC, and miR-30a-5p directly targets the 3′-UTR of CD73 to decrease CD73 expression. **a** Relative miR-30a-5p levels in 59 NSCLC tissues (T) and paired noncancerous lung tissues (N). **b** Scatter diagram showing relative CD73 mRNA expression in NSCLC tumors and adjacent normal lung tissues from a public data set (GSE36681). **c** qRT-PCR analysis of relative miR-30a-5p expression in human NSCLC cell lines. **d** Schematic diagram showing the subcloning of the predicted miR-30a-5p binding site at two positions (328–355 and 1442–1449) of the CD73 3′-UTR into a psiCHECK-2 luciferase construct. Predicted duplex formation between miR-30a-5p and the wild-type or mutant miR-30a-5p binding site is indicated. **e**–**g** Luciferase activity of the construct containing the wild-type or mutant CD73 3′-UTR reporter gene in A549 and H226 cells co-transfected with the negative control (NC) or miR-30a-5p. Scrambled sequences were used as the NC. Relative Renilla luciferase activity was determined and normalized against firefly luciferase activity. **h**–**j** and **k**–**m** Expression of miR-30a-5p and CD73 in NSCLC cells transfected with miR-30a-5p mimics or inhibitor was detected by qRT-PCR and western blot analysis respectively. ^*^ *P* < 0.05; ^**^ *P* < 0.01; ^***^ *P* < 0.001

miR-30a-5p expression is significantly downregulated in NSCLC tissues (Additional file 7: Table S3) [28]. This affects crucial cellular pathways, including cell-cell adhesion and signaling, cell cycle regulation and apoptosis, and plays a significant role in the pathogenesis of NSCLC (Additional file 8: Figure S5). Among the 59 randomly selected paired tissues from NSCLC patients, miR-30a-5p expression was found to be significantly reduced in tumor tissues when compared with paired noncancerous tissues (Fig. 4a, and Additional file 1: Table S1). Further, a public dataset (GSE36681) containing 47 NSCLC tissues and 47 normal lung tissues showed that miR-30a-5p expression was downregulated in human NSCLC tissues (Fig. 4b). Moreover, we examined CD73 mRNA expression in eight NSCLC cell lines (Fig. 4c) and found that miR-30-5p levels in lung squamous carcinoma cell lines (H226 and H520) was lower than in lung adenocarcinoma cell lines in general.

### Ectopic miR-30a-5p reduces CD73 expression by targeting the CD73 3′-UTR in NSCLC cells

We used bioinformatics analysis to identify additional novel targets of miR-30a-5p, and found that miR-30a-5p is one of only two miRNAs that could bind to the 3′-UTR of CD73 mRNA (TargetScanHuman: http://www.targetscan.org/). Therefore, it is possible that miR-30a-5p inhibits CD73 expression by directly binding to its 3′-UTR region (Fig. 4d). To confirm that CD73 expression is directly regulated by miR-30a-5p, we subcloned the CD73 3′-UTR containing the miR-30a-5p binding site (wild type/mutant type) into the psiCHECK-2 vector (Fig. 4e). We transiently cotransfected the reporter construct with the miR-30a-5p mimics or inhibitor into A549 and H226 cells. The results showed that miR-30a-5p significantly inhibited luciferase activity in cells transfected with the wild-type CD73 3′-UTR but did not repress luciferase activity in cells containing the mutant construct (Fig. 4f and g).

In addition, we detected CD73 expression in A549 and H226 cells transfected with miR-30a-5p and its inhibitor, respectively, while the control cells were transfected with miR-NC. The data showed that the expression of miR-30a-5p is increased in NSCLC cells transfected with the miR-30a-5p mimics compared with the cells transfected with miR-NC (Fig. 4h). Moreover, the expression of miR-30a-5p was lower in NSCLC cells transfected with the miR-30a-5p inhibitor than in cells transfected with miR-NC (Fig. 4k), In line with the expression of miR-30a-5p, the level of CD73 was downregulated or upregulated, as determined using qRT-PCR and western blot analysis (Fig. 4i–m).

### Overexpression of miR-30a-5p can inhibit NSCLC cell proliferation and migration

**Fig. 5:**
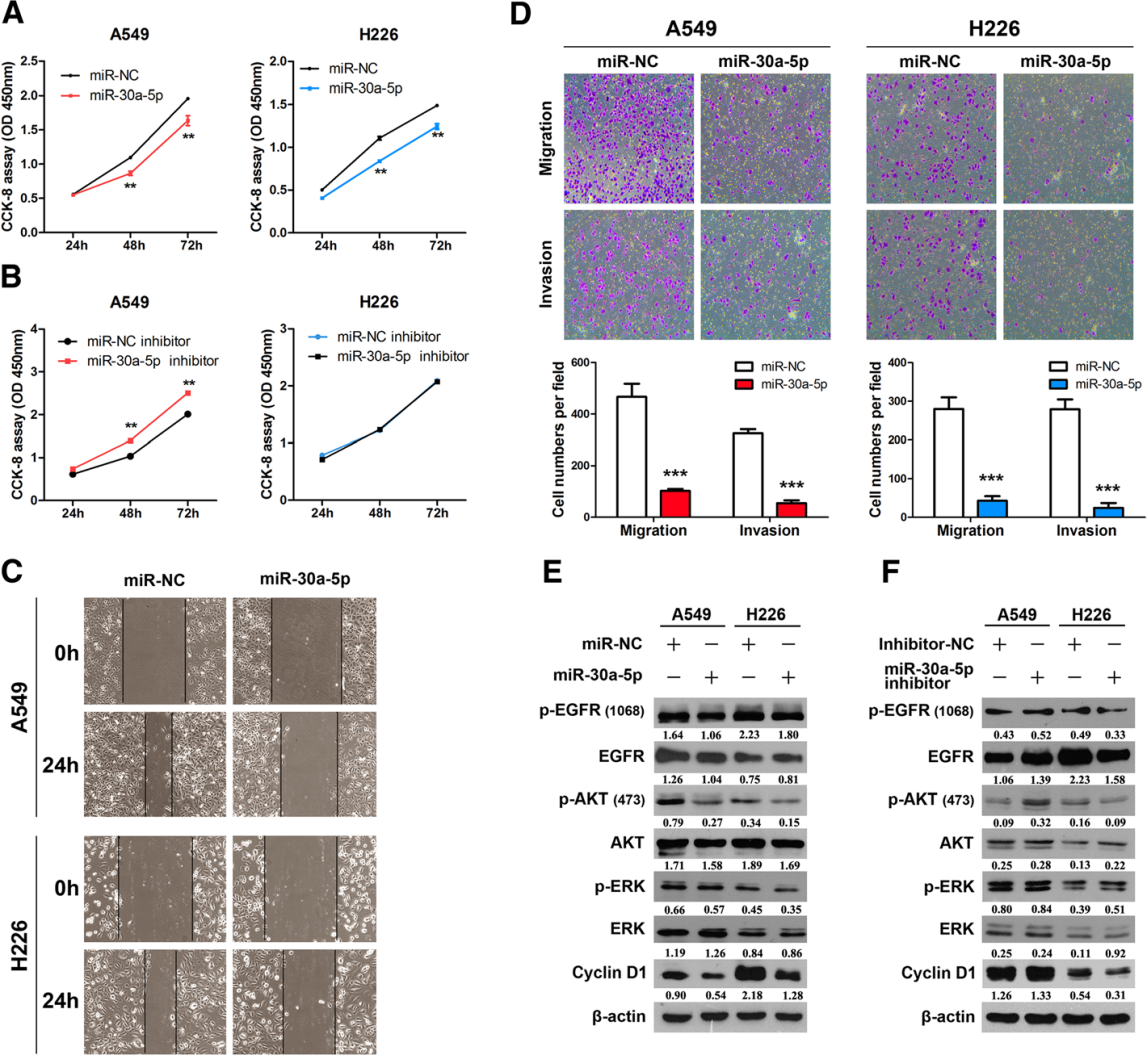
Overexpression of miR-30a-5p inhibits NSCLC cell proliferation and motility. **a** & **b** CCK-8 assay of cell viability in NSCLC cell lines transfected with miR-30a-5p mimics or miR-30a-5p inhibitor at 24, 48 and 72 h. **c** A wound healing assay was performed to observe the role of miR-30a-5p transfection in cells. The data showed that the speed with which the cells migrated towards the scratch was lower in cells transfected with the miR-30a-5p overexpression vector than in the control cells. **d** Overexpression of miR-30a-5p inhibits invasion and migration of NSCLC cells. The A549 and H226 cell lines were transfected with miR-30a-5p mimics and allowed to migrate through 8-μM pore Transwells. The cells that migrated were stained and counted in at least three microscopic fields (magnification, ×100). Then, cells were treated as described before and allowed to invade through the Matrigel-coated membrane in Transwells. The invasive cells were stained and counted under a light microscope. **e** and **f** The A549 and H226 cells were treated with or without miR-30a-5p mimics or the miR-30a-5p inhibitor for 72 h, respectively. The expression levels of p-EGFR, EGFR, p-AKT, AKT, p-ERK, ERK and Cyclin D1 were analyzed by western blotting. ^**^ *P* < 0.01; ^***^ *P* < 0.001

Furthermore, to determine the function of miR-30a-5p in NSCLC, we induced overexpression of miR-30a-5p by using miR-30a-5p mimics and silencing miR-30a-5p using the miR-30a-5p inhibitor in NSCLC cells and studied the effects on cell growth. CCK-8 assays showed that NSCLC cells overexpressing miR-30a-5p had significantly lower proliferation ability than the control cells (Fig. 5a and b). In contrast, silencing of miR-30a-5p resulted in a significantly higher proliferation rate than observed in the control cells, with the exception of the H226 cell lines (Fig. 5b).

The wound healing assay was performed to observe the role of miR-30a-5p transfection in A549 and H226 cells. As shown in Fig. 5c, the speed with which cells migrated towards the scratch was lower in the cells transfected with the miR-30a-5p mimics than in the control cells. Transwell assay of A549 and H226 cells further indicated that loss of miR-30a-5p considerably suppressed the migratory ability of NSCLC cells (Fig. 5d). Taken together, these observations suggest that miR-30a-5p may have tumor suppresser functions in NSCLC.

In line with the results for CD73-silenced cells, our data showed that p-EGFR and p-AKT show significantly lower overexpression in the miR-30a-5p-overexpressing cell lines than in the control cells (Fig. 5e). In contrast, miR-30a-5p silencing led to higher p-AKT expression than in the control cells. These findings were not observed in the H226 cell lines, but this result is in line with the findings of the CCK-8 assay in which the miR-30a-5p inhibitor was used (Fig. 5f).

### CD73 knockdown in NSCLC cells inhibits tumor growth via overexpression of miR-30a-5p in nude mice

**Fig. 6:**
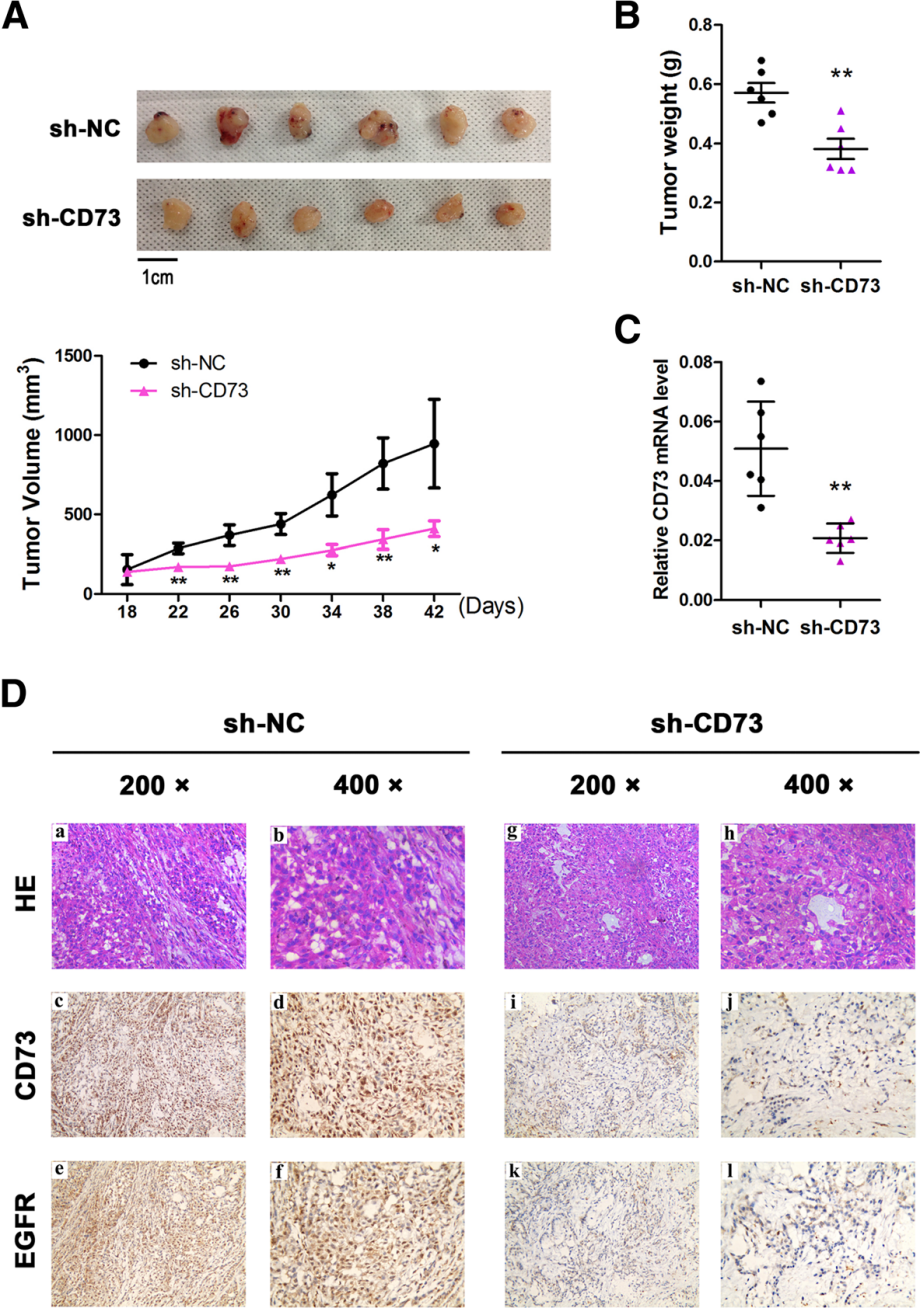
CD73 promotes tumor growth in vivo. **a** CD73-silenced A549 cell xenografts in nude mice (*n* = 6) at the experimental endpoint; tumors were dissected and photographed as shown. Tumor growth curves in mice (*n* = 6 in each group) inoculated with the indicated cells at the indicated days; **b** Each tumor formed was weighed. **c** CD73 mRNA expression in tumors was detected by qRT-PCR analysis. **d** Hematoxylin and eosin (H & E) staining confirmed the presence of tumor cells in slices of the indicated tumor sections. Immunohistochemical staining for CD73 and EGFR was quantified based on staining intensity. ^*^ *P* < 0.05; ^**^ *P* < 0.01

To clarify the cellular mechanisms underlying miR-30a-5p-mediated tumor suppression, control A549 sh-NC cells and the corresponding stable CD73-silenced cells were inoculated into BALB/C athymic mice. As shown in Fig. 6a and b, tumors formed by the CD73-silenced cells were smaller in size and weight than those formed from the control cells. The tissues resected from the xenograft tumors were analyzed to verify CD73 and EGFR expression using qRT-PCR and IHC (Fig. 6c and d).

**Fig. 7:**
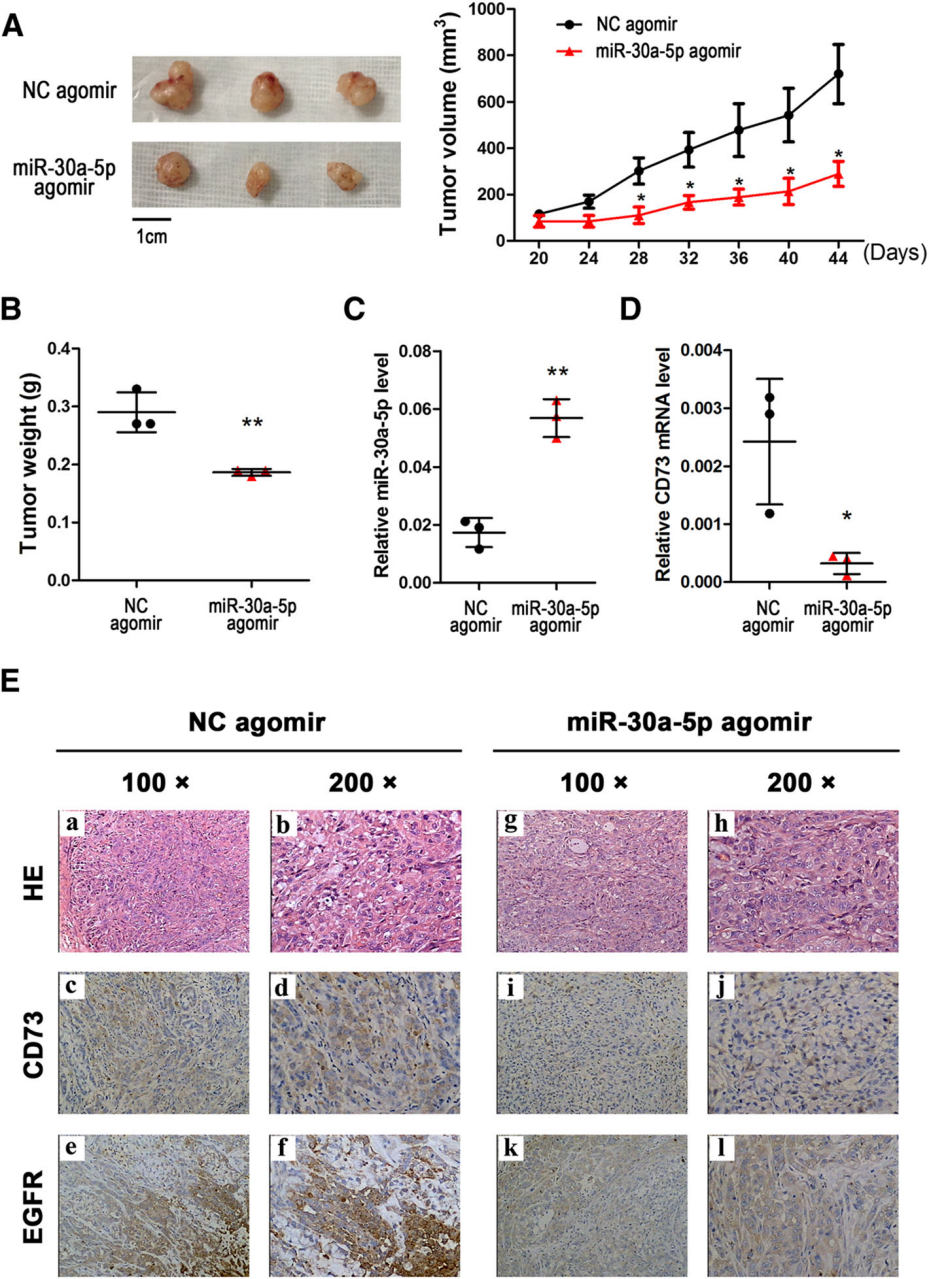
**a** miR-30a-5p inhibits tumor growth by targeted CD73 in vivo. At the experimental endpoint, tumors treated with the miR-30a-5p agomir were dissected and photographed as indicated. Tumor growth of A549 xenografts in nude mice treated with the miR-30a-5p agomir and NC agomir (*n* = 3). The graph shows the tumor growth curves at the time of sacrifice with respect to the first measurements, after the administration of 2 nmol miR-30a-5p agomir or NC agomir per mouse seven times every 4 days; the arrows indicate the weight of the excised tumors (mean ± SD, *n* = 3). **b** Each tumor formed by the indicated cells was weighed. **c** and **d** qRT-PCR analysis of miR-30a-5p levels and CD73 mRNA expression in excised tumors transfected with the miR-30a-5p agomir and NC agomir; U6 and β-actin were used as internal controls, respectively. **e** Hematoxylin and eosin (H & E) staining confirmed the presence of tumor cells in slices of the indicated tumor sections. Immunohistochemical staining for CD73 and EGFR was quantified based on staining intensity. ^*^ *P* < 0.05; ^**^ *P* < 0.01

As miR-30a-5p is significantly downregulated in NSCLC, an miR-30a-5p agomir was used for replacement therapy. As shown in Fig. 7c and d, the miR-30a-5p agomir resulted in a significant increase in the expression of miR-30a-5p and a significant decrease in the expression of CD73, and the tumors treated with the miR-30a-5p agomir were smaller in size and weight than the control tumors (Fig. 7a and b). These findings are consistent with those in the previous paragraph. Moreover, the resected tissues from the treated xenograft tumors were analyzed to verify EGFR and CD73 expression using IHC (Fig. 7e). All these findings indicate that repression of CD73 could inhibit lung cancer cell growth in vivo, and that this mechanism is regulated by miR-30a-5p.

## Discussion

The findings of the present study indicate that CD73 is a critical factor in the tumorigenesis of NSCLC and that its mechanism involves the EGFR signaling pathway. This finding is consistent with the results of studies on other cancer types [11, 32].

miR-30a has been found to be one of the miRNAs that are downregulated in various types of solid tumors, including ovarian cancer, colon cancer, prostate cancer, myeloma cells and lung cancer [34–36]. Further, in an elegant study conducted by Cheng et al., miR-30a was found to be significantly downregulated in the primary tumor of patients with metastatic cancer; this implies that miR-30a has some putative function in cancer and potentially metastasis [37]. However, the tumor suppressive role of miR-30a is poorly understood. In the present study, we have reported for the first time that miR-30a-5p plays an important role in the suppression of cell proliferation, migration and invasion in NSCLC via its effects on CD73 gene expression both in vitro and in vivo.

In the present study, we detected the expression of CD73 in NSCLC tissues and cell lines. CD73 expression was upregulated in NSCLC tissues compared with matched paracancerous tissues. Moreover, we identified the expression of CD73 in the NSCLC cell lines examined, which shows that CD73 is frequently overexpressed in NSCLC. Our findings indicate that shRNA-induced knockdown of CD73 significantly inhibits NSCLC cell proliferation by repressing the NSCLC cell cycle. The co-expression of CD73 and EGFR has been reported in other types of cancers [11, 32]. Consistent with the previous findings, in the present study too, CD73 was found to promote EGFR expression. However, the interactions and underlying mechanisms need to be elucidated. Therefore, our findings demonstrated that CD73 may play a tumor-promoting role in NSCLC via its effects on EGFR signaling.

Although there is ample evidence for the upregulation of CD73 expression in human cancers, the underlying mechanisms are poorly understood. In this study, we focused on NSCLC cells and tissues to determine whether miRNAs can epigenetically influence CD73 expression. Intriguingly, the miR-30a-5p level was found to be reduced in NSCLC cells and tissues, which is in line with the findings of other studies [36, 38]. The results imply that miR-30a-5p functionally contributes to the expression of CD73 in NSCLC. Although several miRNAs, including miR-30a-5p, have been implicated in prostate cancer, myeloma cells and lung cancer, the targets of miR-30a-5p have not yet been identified [36]. Therefore, in the present study, we performed in silico prediction of microRNA targets and found that miR-30a-5p can potentially bind to two target sites of CD73 3′-UTR, and that miR-30a-5p is one of only two such molecules that can bind to the 3′-UTR of CD73 mRNA. Next, we used two methods to confirm whether CD73 is a bona fide target of miR-30a-5p. First, a luciferase reporter assay was performed, and it showed that miR-30a-5p can selectively target two putative site of the CD73 3′-UTR. Second, ectopic expression of miR-30a-5p in NSCLC cells was examined and found to be significantly reduced, and CD73 mRNA and protein expression was found to have increased. This provides a strong rationale for our findings that both low miR-30a-5p and high CD73 expression are found in NSCLC.

It is known that miR-30a-5p is frequently downregulated in human cancer tissues [35, 38] as well as in human epithelia-derived cancer cells [37]. Our present data showed that CD73 is required for NSCLC cell proliferation (Fig. 2). These findings together seemed to indicate the possibility that miR-30a-5p may inhibit NSCLC cell proliferation. As expected, ectopic miR-30a-5p expression was found to inhibit NSCLC cell proliferation and migration in NSCLC cells, which further confirms the tumor-promoting function of CD73. However, we cannot exclude the possibility that miR-30a-5p may have different functions, may be even tumor-inhibiting functions, in other types of cancer, because a single miRNA may have different functions in different cellular contexts [39].

A considerable amount of evidence suggests that miRNAs play a role in fighting drug resistance. Overexpression of CD73 is also associated with resistance to antitumor agents. Further, some studies have shown the therapeutic potential of CD73 blockade for cancer therapy [40–42], and the effects of CD73 on tumor cells via the EGFR signaling pathway [11, 32]. In keeping with all these previous findings, we found that shRNA-mediated CD73 knockdown increased the sensitivity of NSCLC cells to gefitinib and lapatinib.

It was recently reported that EGFR signaling is coupled with activation of cap-dependent translation in NSCLC cells expressing wild-type EGFR [11]. Resistance to EGFR-TKI can be mediated through multiple signaling pathways that converge upon cap-dependent translation in NSCLC cells expressing wild-type EGFR. Interestingly, our present study showed that CD73 affected the efficacy of EGFR-targeted therapies in NSCLC cells with wild-type EGFR. Thus, future investigation into the molecular mechanisms of CD73-mediated drug resistance could help develop novel CD73-based therapeutic agents to improve the treatment of NSCLC.

## Conclusions

Taken together, these results are the first to report that CD73 expression is upregulated in NSCLC and is correlated with a decrease in miR-30a-5p expression. Further, we found that miR-30a-5p inhibits CD73 expression by directly targeting CD73 3′-UTR, and thereby represses NSCLC cell proliferation. Thus, our findings shed light on the mechanistic interaction between miR-30a-5p and CD73 in NSCLC carcinogenesis. This miR-30a-5p-mediated downregulation of CD73 provides new insight into therapy strategies for NSCLC.

## Methods

### Tissue samples

Fifty-nine paired NSCLC tissues and adjacent noncancerous lung tissues were collected, with the informed consent of the patients from the First Affiliated Hospital of Soochow University between 2009 and 2013. The patients had been diagnosed with NSCLC based on their histological and pathological characteristics according to the Revised International System for Staging Lung Cancer. They had not undergone chemotherapy or radiotherapy prior to tissue sampling. The tissue samples were snap-frozen and stored in a cryofreezer at −80 °C. This study was approved by the Academic Advisory Board of Soochow University.

### Cell culture

Human NSCLC cell types A549, H1650 and SPC-A1 (lung adenocarcinoma cell lines) and H460 (giant-cell lung carcinoma cell line), 95C, 95D and H226, H520 (lung squamous carcinoma cell line) were purchased from the Cell Bank of the Chinese Academy of Sciences (Shanghai, China). The cells were grown in RPMI 1640 medium containing 10% fetal bovine serum (FBS) (Gibco, Carlsbad, CA, USA) and l-glutamine (Invitrogen, Carlsbad, CA, USA) at 37 °C in a humidified atmosphere containing 5% CO_2_. Genetic characteristics of the cells were determined by the Beijing Microread Genetics company using a Goldeneye™ 20A Kit and ABI 3100. All cell lines were passaged for less than 3 months and tested in January 2016.

### Immunohistochemical assay

Immunohistochemical (IHC) analyses of tissues were conducted as described in our previous study [43]. In brief, sections were incubated with EGFR (A-10) and CD73 (IE9) specific monoclonal primary antibodies (diluted 1:100; Santa Cruz Biotechnology, Santa Cruz, CA, USA) overnight at 4 °C, then incubated with biotinylated secondary antibodies. The reactions were developed using the DAB Kit (BD Bioscience, San Jose, CA, USA) and the sections were counterstained with hematoxylin.

### RNA extraction and quantitative real-time PCR analysis

RNA isolation, cDNA synthesis and quantitative reverse transcription–PCR analysis were performed as previously described [28]. The primer sequences used for CD73 mRNA detection were 5′-TCTTCTAAACAGCAGCATTCC-3′ (forward) and 5′-CATTTCATCCGT GTGTCTCAG-3′ (reverse). Ct values for CD73 mRNA and miR-30a-5p were equilibrated to *ACTB* mRNA and U6, respectively, which were used as internal controls. The △△Ct method was applied to calculate the relative expression of these proteins.

### Kinase and Western blot assays

5′-(N-Ethylcarboxamido) adenosine (NECA, E2387) and Adenosine 5′-(α, β-methylene) diphosphate (APCP, M3763) were purchased from Sigma. Cells were seeded into 6-well plates at a concentration of 30 × 10^4^ cells/well. After 24 h, cells were treated with NECA (1 μM) for 24 h or APCP (10 μM) for 1 h, then were harvested and lysed in RIPA buffer (Cell Signaling Technology, Danvers, MA, USA) containing a protease and phosphatase inhibitor cocktail (Sigma-Aldrich, St. Louis, MO, USA).

For the human receptor tyrosine kinase (RTKs) assay, we used the phosphorylation antibody array-AAH-PRTK-1 (RayBiotech Inc.). Protein lysates were incubated with the array membrane and protein signal was visualized using a chemifluorescence detection system (Bio-Rad) according to the manufacturer’s protocol. Relative density of specific protein expression was determined using Quantity One software.

Western blot analysis was performed as previously described [43]. The antibodies used in the analysis were anti-CD73 (D7F9A), anti-pEGFR (Tyr1068, 1H12), anti-pAKT (Ser473, D9E), anti-AKT, anti-pERK (Thr202/204, D13.12.4E), anti-ERK (137 F5), anti-pFak (Tyr397, D20B1), anti-FAK (D2R2E) and anti-CyclinD1 (92G2, all from Cell Signaling Technology, Danvers, MA, USA), anti-EGFR (A-10, Santa Cruz, CA, USA), anti-β-actin and anti-mouse or anti-rabbit secondary antibodies (Cell Signaling Technology).

### Plasmids construction, transient transfection and luciferase assay

To construct a plasmid containing the CD73 3′-untranslated region (3′-UTR) fused to the 3′-end of a luciferase reporter, 1746-bp sequences containing the predicted miR-30a-5p target sites were synthesized and ligated into the pGL3-control vector (Promega, Madison, WI, USA). CD73 3′-UTR was amplified with the primers 5′-GGCTAG*TCTAGA*CTGCCTTTTAGGACCTGGCT-3′ (forward) and 5′-GGCTAG*TCTAGA* ACCGAGGCTATTATTTTGCTGC-3′ (reverse) and were subcloned into a pGL3 control vector with the restriction endonuclease *Xba*I site (italic font) to generate pGL3-CD73-3′-UTR. The 3′-UTR of CD73 containing two putative miR-30a-5p-binding sites was amplified and cloned into a pGL3-control vector separately. In the mutated fragment, four mutational bases were introduced into the predicted miR-30a-5p target sites. The wild-type and mutated fragments were directly synthesized (Genewiz, Suzhou, China), digested with *Xba*I and subcloned into the pGL3-control vector. Subsequently, cells were seeded into 24-well plates and co-transfected with the constructed plasmid, the pRL-TK plasmid and with either miR-30a-5p mimics or miR-NC; miR-30a-5p inhibitor and matched NC were purchased from RiboBio Co., Ltd (Guangzhou, China). After 48 h, cells were collected and their luciferase activity was measured using the Dual-Luciferase Reporter Assay Kit (Promega). The results are expressed as the relative firefly luciferase activity, which is obtained after normalization to Renilla luciferase activity. All the transient transfections, including the miR-30a-5p mimic, inhibitor and miR-NC transfections, were performed using Lipofectamine 2000 (Invitrogen, Carlsbad, CA, USA).

### Establishment of CD73-silenced stable cell lines

To establish stable cell lines with silenced CD73 expression, two DNA fragments (CD73 shRNA-1, 5′-GATCCGGAATCGTTGGATACACTTCCTTCAAGAGAGGAAGTG TATCCAACGATTCCTTTTTTG-3′; CD73 shRNA-2, 5′-GATCCGCCGCTTTAGAGAATG CAACATTCAAGAGATGTTGCATTCTCTAAAGCGGCTTTTTTG-3′) were subcloned into the lentiviral vector pGLV2-U6-Puro (GenePharma, Shanghai, China) containing the endonucleases *Age*I and *Eco*RI. A scrambled sequence (underscored) of CD73 shRNA, which served as the negative control, was used: 5′-GATCCGTTCTCCGAACGTGTCACGTT TCAAGAGAACGTGACACGTTCGGAGAACTTTTTTG-3′. Then, the CD73-silenced construct or negative control was co-transfected with packaging plasmids into human embryonic kidney 293 T cells using Lipofectamine 2000 (Invitrogen). After 48 h, cells were infected with the packaged lentiviruses and cultured for 2 days before being selected with 0.4 μg/ml and 2 μg/ml of puromycin, respectively (Sigma-Aldrich, St Louis, MO, USA).

### Cell proliferation analysis and drug treatment

Cell proliferation was examined using Cell Counting Kit-8 (Boster, Wuhan, China). Cells or the corresponding negative control cells were seeded in 96-well plates at a density of 2 × 10^3^ cells per well and further grown under normal culture conditions for 24, 48 and 72 h. Cell viability was determined according to the manufacturer’s instructions. The experiment was performed in triplicate. We also detected cell proliferation using a clonogenic assay. In brief, cells transfected with miR-30a-5p mimics and sh-CD73 or sh-NC were suspended in complete culture medium and 200 cells were reseeded into a 60 mm plate. After incubation for 14 – 20 days, foci of least 50 cells were stained with Giemsa and counted. Cell viability was measured according to manufacturer’s instructions at several time points (24, 48 and 72 h). Each experiment was performed in triplicate. For drug treatment, stable CD73-knockdown cells were plated into 96-well plates, and gefitinib and laptinib (gefitinib C#s1025, laptinib C#s2111, Selleck Chemicals, Houston, TX, USA) were added to the cultures. Cell viability was assessed 72 h after the drug treatment.

### Wound healing, migration and invasion assays

The motility of cells was analyzed as previously described [43]. For the wound healing assay, cells were seeded into 6-well plates and cultured for 48 h to form a monolayer. The monolayer was then scratched with a new 10-μl pipette tip across the center of the well, washed gently twice with PBS and replenished with fresh medium. The cells were grown for an additional 24 h and were visualized on a microscope (CKX41, Olympus).

For the migration assay, 5 × 10^4^ cells in medium containing 1% FBS were seeded into the upper chamber of a Transwell insert, 800 μl medium containing 10% FBS was into the lower chamber and then incubated at 37 °C for 24 h, according to the manufacturer’s instructions. For the invasion assay, the inserts were coated with the Matrigel matrix (BD Science, Sparks, MD, USA) and cells were diluted in serum-free medium before plating and incubated at 37 °C for 2 h. In both assays, the cells were then photographed and counted.

### Cell cycle analysis

According to the instructions of the Cell Cycle Analysis kit (Beyotime, Shanghai, China), cells were cultured in 6-well plates. The cells were collected, washed with cold PBS, fixed in 70% ethanol at 4 °C for 24 h, washed with cold PBS again, and stained with a propidium iodide (PI)/RNase A mixture. The cells were then kept in the dark at 37 °C for 30 min and analyzed with a fluorescence-activated cell sorting (FACS) caliber system (Beckman Coulter, Brea, California).

### Animal experiments and immunocytochemistry staining

Female BALB/c athymic nude mice (4–6 weeks old and weighing 16–20 g) were purchased from the Experimental Animal Center of Soochow University and bred under pathogen-free conditions. All the animal experiments were carried out in accordance with the Guide for the Care and Use of Experimental Animals Center of Soochow University. To establish the lung carcinoma xenograft model, A549 cells and stable CD73-knockdown A549 cells were suspended in 100 ml PBS and inoculated subcutaneously into the flanks of nude mice. After 8–10 days, the nude mice with transplanted A549 cells were randomly divided into two groups (8 mice in each group). The mice with transplanted CD73-knockdown A549 cells were also split into two groups. An miR-30a-5p agomir and NC agomir (RiboBio Co. Ltd., Guangzhou, China) were directly injected into the A549 implanted tumors at a dose of 2 nmol (in 20 μl PBS) per mouse every 4 days seven times (28 days total). Chemically stabilized miRNAs may have markedly improved pharmacological properties, as described before [44]. Tumor volume (*V*) was determined by measuring the length (*L*) and width (*W*) with a vernier caliper and applying the formula *V* = (*L* × W^2^) × 0.5.

### Statistical analysis

Differences in CD73 and miR-30a-5p expression between NSCLC tissues (T) and adjacent noncancerous lung tissues (N) were analyzed using a paired *t*-test (two-tailed). For cell lines, differences between two groups were assessed using an unpaired *t*-test (two-tailed). The clinicopathologic characteristics and expression levels of mRNA and miRNA in the NSCLC samples were compared using nonparametric tests (Mann–Whitney U-test for comparison between two groups, and the Kruskall-Wallis test for comparison between three or more groups). Two-way ANOVA was used to determine the difference in cell growth between two groups. Differences were considered to be significant at *P* < 0.05. All statistical analyses were performed using GraphPad Prism 5.02 (GraphPad, San Diego, CA, USA) and the SPSS 16.0 software (SPSS, Chicago, IL, USA).

## Additional files

Additional file 1: Table S1. Demographic and clinical characteristics of NSCLC patients and the level of miR-30a-5p and CD73 mRNA expression in tumor tissue specimens. (DOC 39 kb)
Additional file 2: Figure S1. The expression of CD73 in stable cell lines. (A) CD73 mRNA and (B) protein levels in cell lines stably transfected with two CD73 shRNAs (sh-CD73-1 and sh-CD73-2) or negative control (sh-NC). Scrambled sequence was used as sh-NC. (TIF 394 kb)
Additional file 3: Figure S2. Human RTK phosphorylation array. The specific kinase target of CD73 was screened using a human RTK phosphorylation array, including 71 RTKs, with both CD73 silenced and control A549 cells. The results showed that phosphorylation of the EGFR family was downregulated in both CD73 silenced cells compared with control. (TIF 613 kb)
Additional file 4: Table S2. Signal densities of human RTK phosphorylation array. (DOCX 20 kb)
Additional file 5: Figure S3. Effects of the CD73 inhibitor APCP and the adenosine analogue NECA in NSCLC cell lines. Cells were seeded into 6-well plates at a concentration of 30 × 10^4^ cells/well, and were treated with NECA (1 μM) for 24 h or APCP (10 μM) for 1 h, before being harvested and lysed in RIPA buffer. The results showed that inhibitor APCP functioned similarly to sh-CD73 suppression in A549 cell lines, but had no effect in H226 cell lines. In line with this result, exogenous adenosine analogue NECA increased EGFR signaling in NSCLC cell lines. (TIF 374 kb)
Additional file 6: Figure S4. The sensitivity of NSCLC cells which transfected with Mock and sh-NC to gefitinib or lapatinb. Mock transfected cells were treated without vector or plasmid. The drug treatment control had drug replaced by DMSO alone. (TIF 386 kb)
Additional file 7: Table S3. Differential expression of miRNAs and miR-30a-5p is downregulated in NSCLC. (DOC 57 kb)
Additional file 8: Figure S5. Pathway and Gene Ontology Affected. Pathway analysis was used to identify the significant pathway of the differential genes according to the KEGG database. The Fisher’s exact test was used to select significant pathways, with the threshold of significance defined by p-value and FDR. Fisher’s exact test was applied to identify the significant GO categories and FDR was used to correct the *P*-value. GO, gene ontology; BP, biological process; MF, molecular function; CC, cellular component. (TIF 3.3 mb)

## Abbreviations

ADP: Adenosine diphosphate
ATP: Adenosine triphosphate
CD73: Ecto-5′-nucleotidase
IHC: Immunohistochemical
microRNA: miRNA
NSCLC: Non-small cell lung cancer
qRT-PCR: Quantitative reverse transcription–PCR
shRNAs: Short hairpin RNAs
