# Supplementary material for: CD73/NT5E is a target of miR-30a-5p and plays an important role in the pathogenesis of non-small cell lung cancer
Source: Mol Cancer. 2017 Feb 3;16:34. doi: 10.1186/s12943-017-0591-1 (PMC5291990; doi:10.1186/s12943-017-0591-1)
Supplement: Additional file 1: Table S1. — Demographic and clinical characteristics of NSCLC patients and the level of miR-30a-5p and CD73 mRNA expression in tumor tissue specimens. (DOC 39 kb) [file 12943_2017_591_MOESM1_ESM.doc]

Table 1. Demographic and clinical characteristics of NSCLC patients and the level of miR-30a-5p and CD73 mRNA expression in tumor tissue specimens

| Characteristics | Number of cases (%) | miR-30a-5p | *P* value | CD73 mRNA | *P* value |
| --- | --- | --- | --- | --- | --- |
| Age (years) |  |  |  |  |  |
| >64 | 35 (59.3%) | 0.0091 ± 0.0032 | 0.403 | 0.0430 ± 0.0205 | 0.401 |
| ≤64 | 24 (40.7%) | 0.0144 ± 0.0058 |  | 0.0703 ± 0.0248 |  |
| Gender |  |  |  |  |  |
| Male | 36 (61.0%) | 0.0100 ± 0.0036 | 0.596 | 0.0584 ± 0.0227 | 0.738 |
| Female | 23 (39.0%) | 0.0133 ± 0.0053 |  | 0.0475 ± 0.0199 |  |
| Histological features |  |  |  |  |  |
| Adenocarcinoma | 32 (54.2%) | 0.0080 ± 0.0034 | 0.272 | 0.0591 ± 0.0228 | **0.029** |
| Squamous cell carcinoma | 21 (35.6%) | 0.0159 ± 0.0060 |  | 0.0495 ± 0.0273 |  |
| Others | 6 (10.2%) | 0.0122 ± 0.0113 |  | 0.0445 ± 0.0249 |  |
| Smoker |  |  |  |  |  |
| No | 31 (52.5%) | 0.0110 ± 0.0041 | 0.914 | 0.0435 ± 0.0154 | 0.483 |
| Yes | 28 (47.5%) | 0.0116 ± 0.0046 |  | 0.0659 ± 0.0286 |  |
| Clinical stage |  |  |  |  |  |
| I +II | 31 (52.5%) | 0.0114 ± 0.0042 | 0.959 | 0.0460 ± 0.0195 | 0.592 |
| III + IV | 28 (47.5%) | 0.0115 ± 0.0044 |  | 0.0632 ± 0.0255 |  |
| Lymph node |  |  |  |  |  |
| No | 34 (57.6%) | 0.0111 ± 0.0038 | 0.961 | 0.0438 ± 0.0179 | 0.447 |
| Yes | 25 (42.4%) | 0.0114 ± 0.0050 |  | 0.0683 ± 0.0284 |  |

Data are presented as mean ± SEM. Kruskal-Wallis test for comparison between three or more groups.
