# Supplementary figures and images for: CD73/NT5E is a target of miR-30a-5p and plays an important role in the pathogenesis of non-small cell lung cancer
Source: Mol Cancer. 2017 Feb 3;16:34. doi: 10.1186/s12943-017-0591-1 (PMC5291990; doi:10.1186/s12943-017-0591-1)

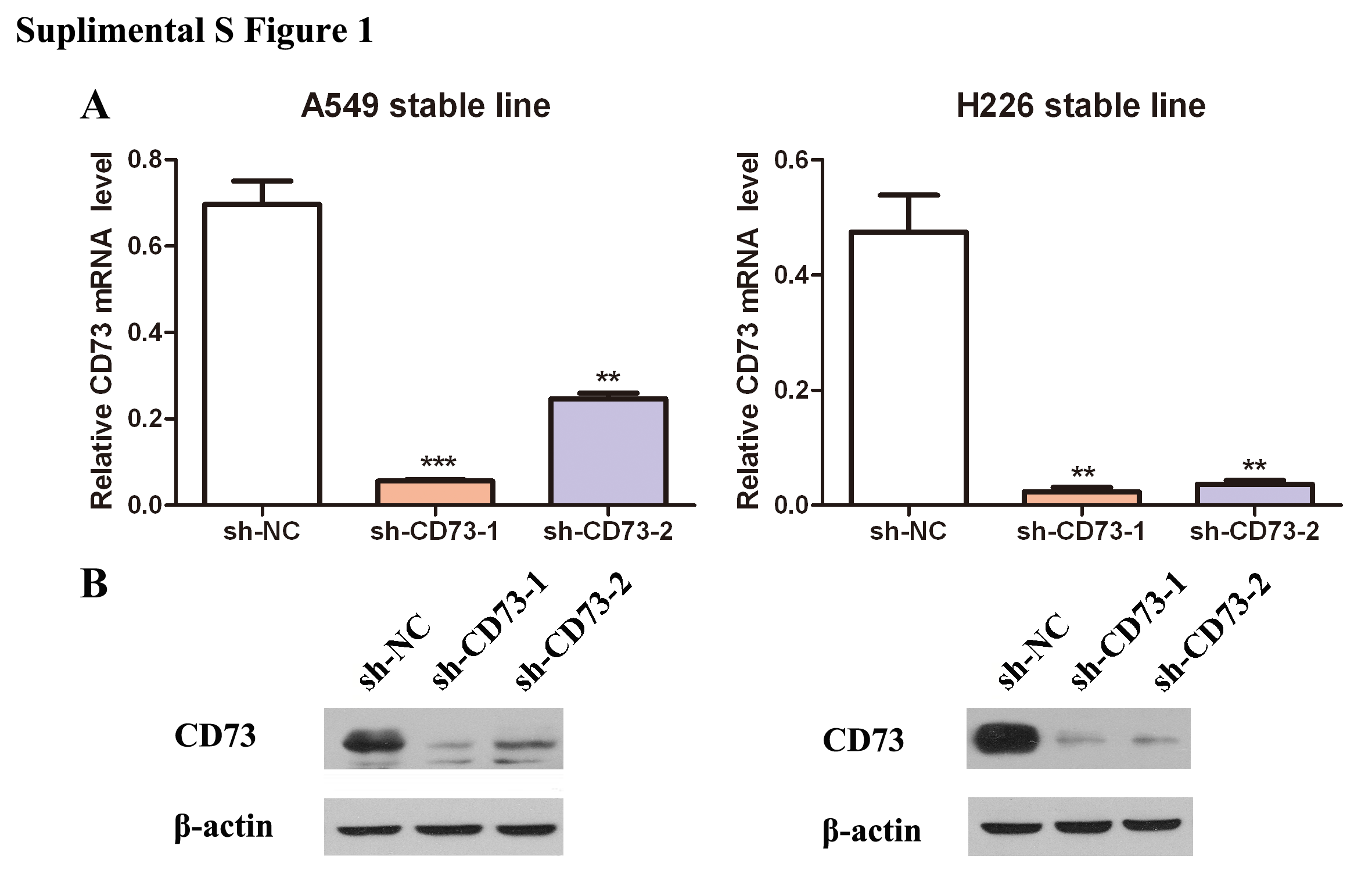

Supplement: Additional file 2: Figure S1. — The expression of CD73 in stable cell lines. (A) CD73 mRNA and (B) protein levels in cell lines stably transfected with two CD73 shRNAs (sh-CD73-1 and sh-CD73-2) or negative control (sh-NC). Scrambled sequence was used as sh-NC. (TIF 394 kb) [file 12943_2017_591_MOESM2_ESM.tif]

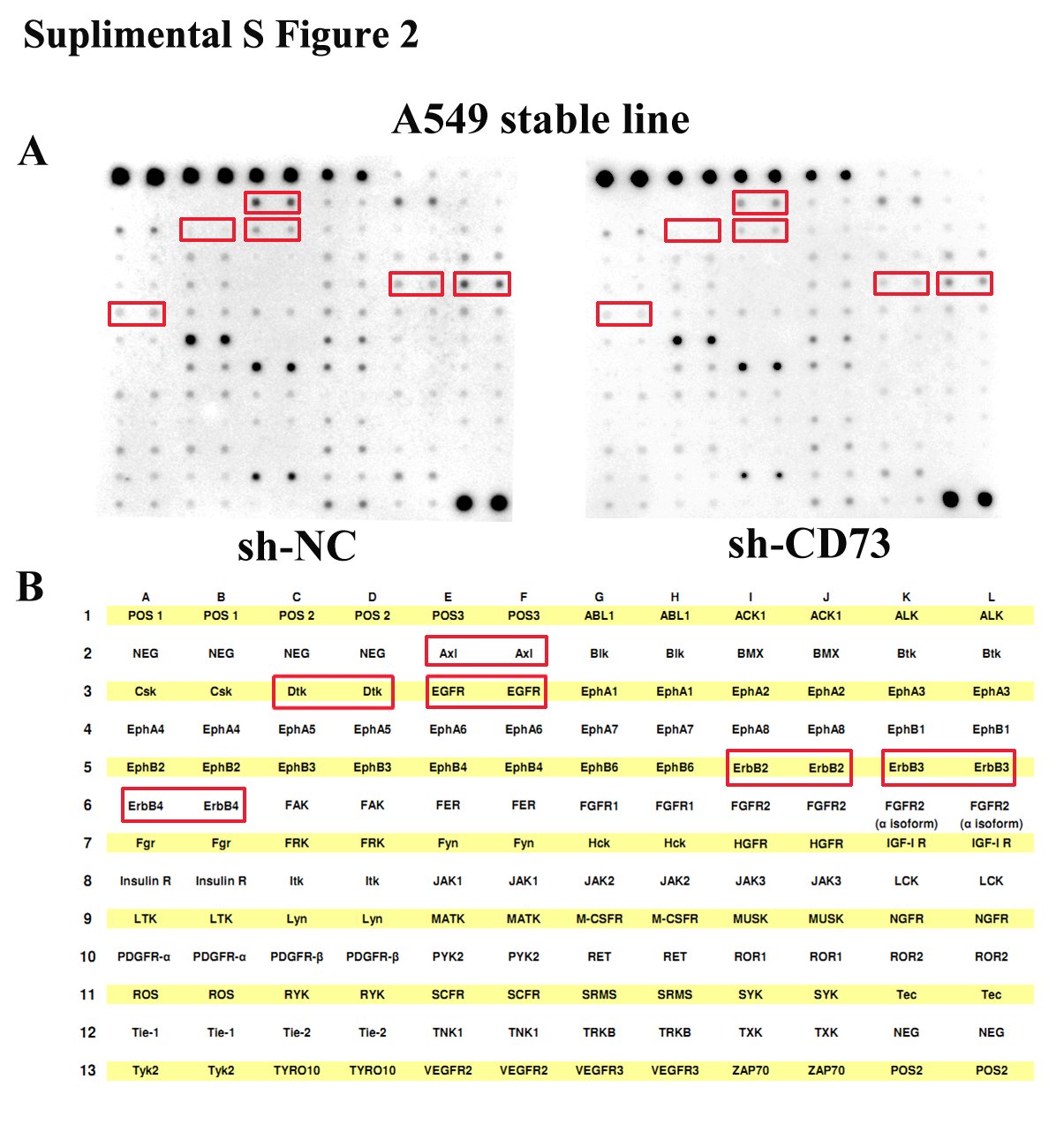

Supplement: Additional file 3: Figure S2. — Human RTK phosphorylation array. The specific kinase target of CD73 was screened using a human RTK phosphorylation array, including 71 RTKs, with both CD73 silenced and control A549 cells. The results showed that phosphorylation of the EGFR family was downregulated in both CD73 silenced cells compared with control. (TIF 613 kb) [file 12943_2017_591_MOESM3_ESM.tif]

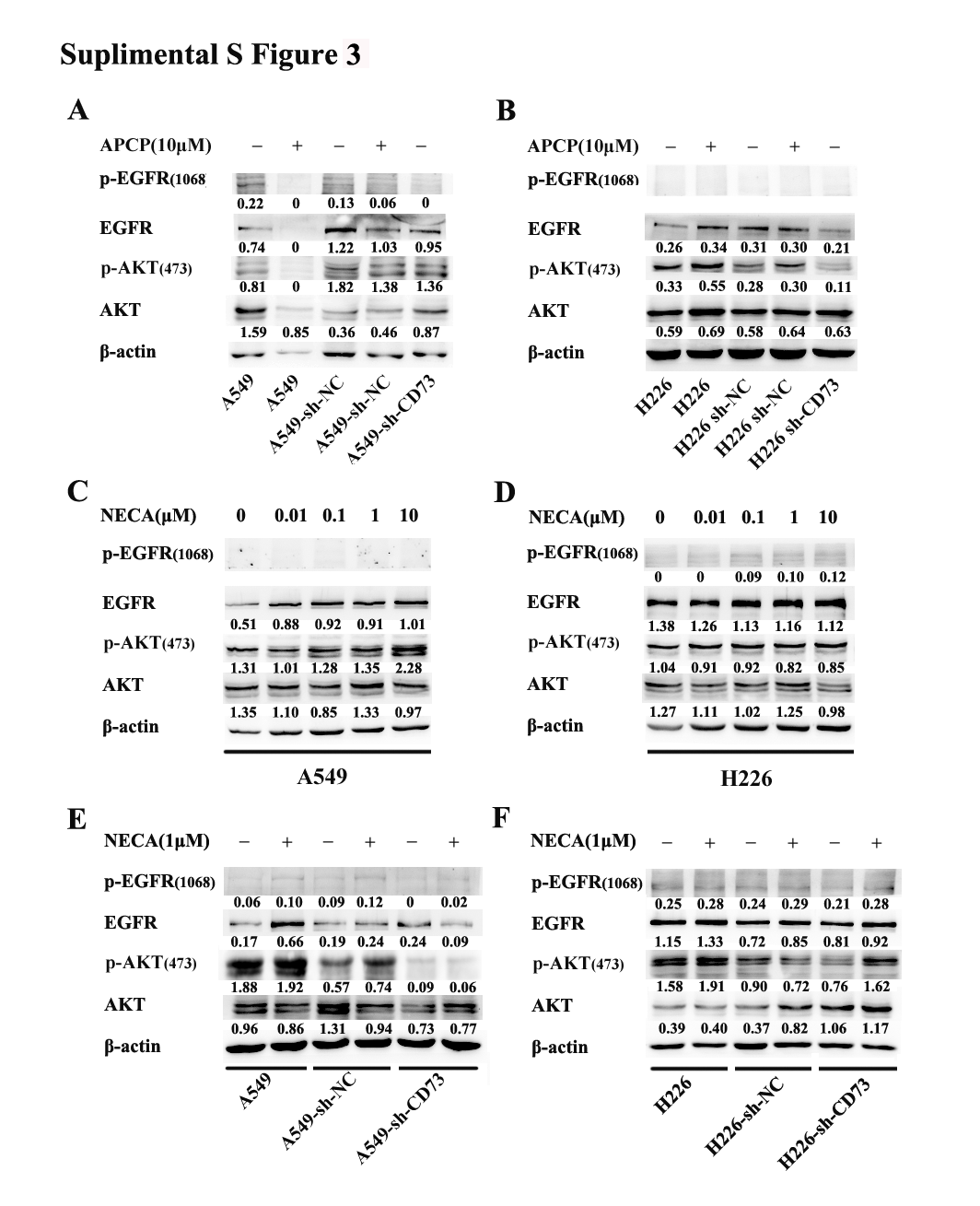

Supplement: Additional file 5: Figure S3. — Effects of the CD73 inhibitor APCP and the adenosine analogue NECA in NSCLC cell lines. Cells were seeded into 6-well plates at a concentration of 30 × 104 cells/well, and were treated with NECA (1 μM) for 24 h or APCP (10 μM) for 1 h, before being harvested and lysed in RIPA buffer. The results showed that inhibitor APCP functioned similarly to sh-CD73 suppression in A549 cell lines, but had no effect in H226 cell lines. In line with this result, exogenous adenosine analogue NECA increased EGFR signaling in NSCLC cell lines. (TIF 374 kb) [file 12943_2017_591_MOESM5_ESM.tif]

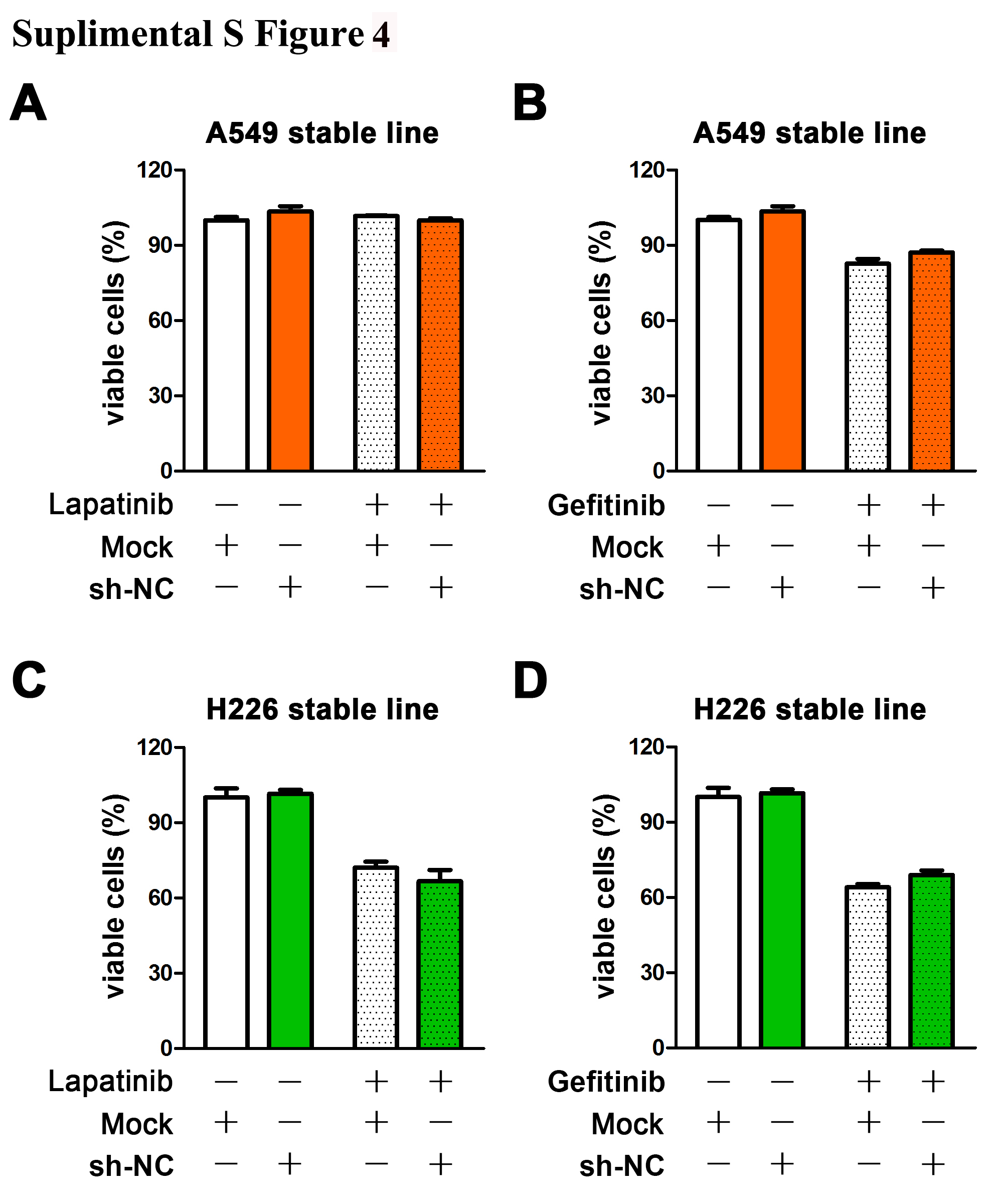

Supplement: Additional file 6: Figure S4. — The sensitivity of NSCLC cells which transfected with Mock and sh-NC to gefitinib or lapatinb. Mock transfected cells were treated without vector or plasmid. The drug treatment control had drug replaced by DMSO alone. (TIF 386 kb) [file 12943_2017_591_MOESM6_ESM.tif]

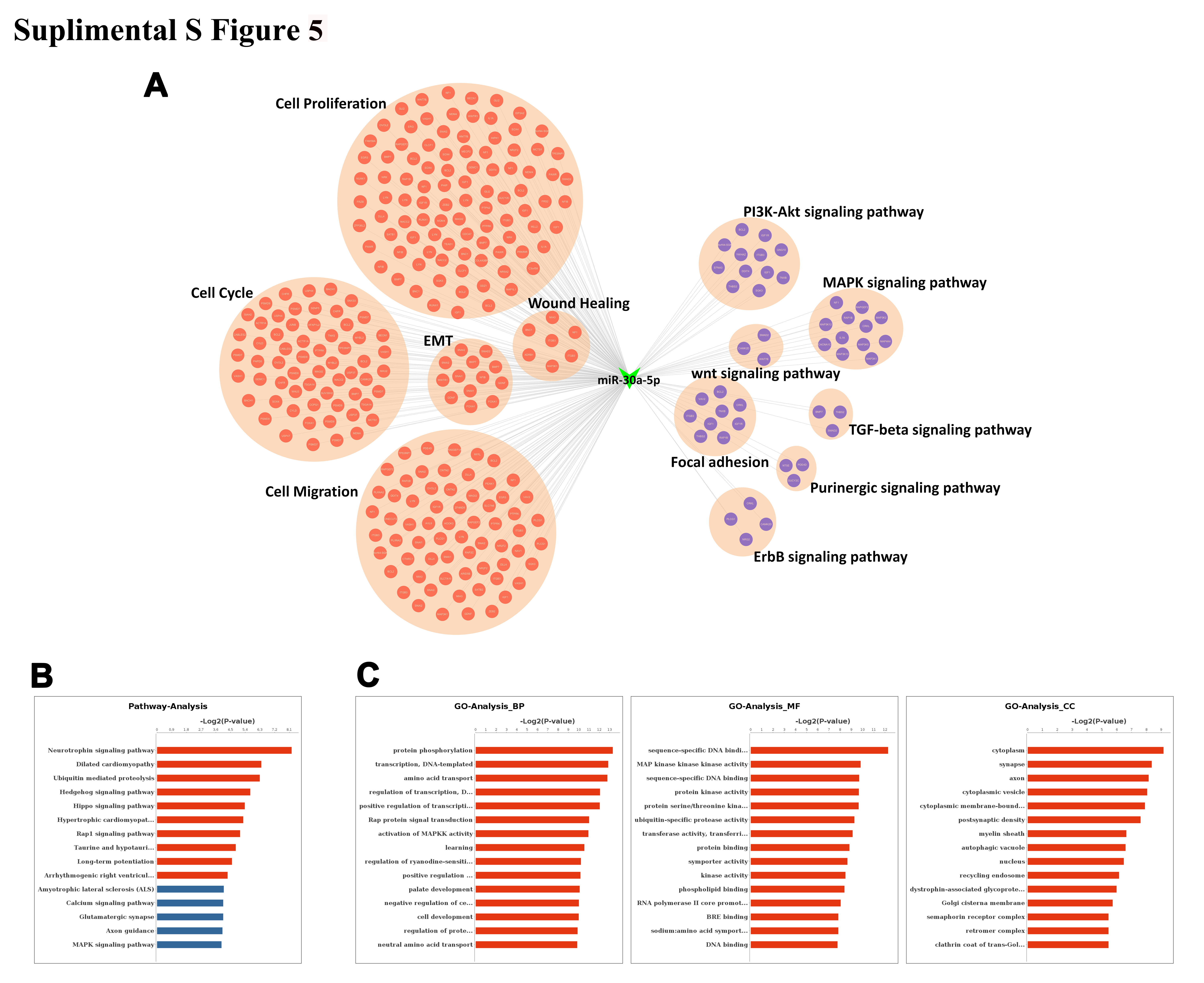

Supplement: Additional file 8: Figure S5. — Pathway and Gene Ontology Affected. Pathway analysis was used to identify the significant pathway of the differential genes according to the KEGG database. The Fisher’s exact test was used to select significant pathways, with the threshold of significance defined by p-value and FDR. Fisher’s exact test was applied to identify the significant GO categories and FDR was used to correct the P-value. GO, gene ontology; BP, biological process; MF, molecular function; CC, cellular component. (TIF 3.3 mb) [file 12943_2017_591_MOESM8_ESM.tif]
