# Supplementary material for: CD73/NT5E is a target of miR-30a-5p and plays an important role in the pathogenesis of non-small cell lung cancer
Source: Mol Cancer. 2017 Feb 3;16:34. doi: 10.1186/s12943-017-0591-1 (PMC5291990; doi:10.1186/s12943-017-0591-1)
Supplement: Additional file 4: Table S2. — Signal densities of human RTK phosphorylation array. (DOCX 20 kb) [file 12943_2017_591_MOESM4_ESM.docx]

**Supplementary Table 2**. List of signal densities of human RTK phosphorylation Antibody Array

| RTKs List |  | A549 | |  | Fold Change |
| --- | --- | --- | --- | --- | --- |
|  |  | sh-NC | sh-CD73 |  | sh-CD73 / sh-NC |
| Dtk |  | 19.00 | 8.48 |  | 0.447 |
| EphB4 |  | 5.00 | 2.68 |  | 0.536 |
| Insulin R |  | 8.00 | 4.47 |  | 0.558 |
| LCK |  | 54.00 | 32.60 |  | 0.604 |
| Fyn |  | 10.00 | 6.70 |  | 0.670 |
| Tie-2 |  | 58.50 | 43.76 |  | 0.748 |
| **ErbB2** |  | 144.50 | 108.51 |  | **0.751** |
| EphA3 |  | 76.00 | 57.60 |  | 0.758 |
| PDGFR-α |  | 31.00 | 23.67 |  | 0.763 |
| NGFR |  | 69.00 | 53.14 |  | 0.770 |
| Tie-1 |  | 95.50 | 74.57 |  | 0.781 |
| EphA5 |  | 134.50 | 105.83 |  | 0.787 |
| **ErbB4** |  | 111.00 | 87.52 |  | **0.788** |
| Axl |  | 449.00 | 355.00 |  | 0.791 |
| Btk |  | 50.50 | 40.19 |  | 0.796 |
| EphA4 |  | 61.00 | 51.35 |  | 0.842 |
| ROS |  | 159.50 | 134.41 |  | 0.843 |
| FAK |  | 122.00 | 106.28 |  | 0.871 |
| LTK |  | 133.50 | 118.78 |  | 0.890 |
| ROR1 |  | 1.00 | 0.89 |  | 0.893 |
| RYK |  | 156.00 | 141.11 |  | 0.905 |
| **ErbB3** |  | 412.50 | 376.43 |  | **0.913** |
| FGFR2 (α isoform) |  | 144.00 | 131.73 |  | 0.915 |
| TYRO10 |  | 68.50 | 62.96 |  | 0.919 |
| EphB3 |  | 131.50 | 121.01 |  | 0.920 |
| **EGFR** |  | 117.00 | 108.96 |  | **0.931** |
| ROR2 |  | 60.50 | 57.16 |  | 0.945 |
| Lyn |  | 168.00 | 159.41 |  | 0.949 |
| Blk |  | 98.50 | 93.77 |  | 0.952 |
| FGFR1 |  | 164.00 | 156.29 |  | 0.953 |
| Csk |  | 261.00 | 249.17 |  | 0.955 |
| FRK |  | 1389.00 | 1361.05 |  | 0.980 |
| ALK |  | 36.00 | 35.28 |  | 0.980 |
| PDGFR-β |  | 14.50 | 14.29 |  | 0.985 |
| IGF-I R |  | 51.50 | 51.35 |  | 0.997 |
| FGFR2 |  | 97.00 | 97.35 |  | 1.004 |
| FER |  | 126.50 | 127.26 |  | 1.006 |
| VEGFR3 |  | 326.00 | 334.46 |  | 1.026 |
| TNK1 |  | 629.00 | 647.03 |  | 1.029 |
| BMX |  | 370.50 | 383.13 |  | 1.034 |
| EphA1 |  | 60.00 | 62.07 |  | 1.034 |
| VEGFR2 |  | 14.50 | 15.18 |  | 1.047 |
| Tyk2 |  | 123.50 | 129.50 |  | 1.049 |
| Tec |  | 19.00 | 20.09 |  | 1.058 |
| Itk |  | 216.50 | 230.86 |  | 1.066 |
| Fgr |  | 66.00 | 70.55 |  | 1.069 |
| ACK1 |  | 101.00 | 108.06 |  | 1.070 |
| JAK2 |  | 321.50 | 344.28 |  | 1.071 |
| ABL1 |  | 2515.00 | 2732.81 |  | 1.087 |
| EphB1 |  | 192.00 | 210.32 |  | 1.095 |
| MATK |  | 94.00 | 104.49 |  | 1.112 |
| EphA6 |  | 19.50 | 21.88 |  | 1.122 |
| EphB6 |  | 134.00 | 150.93 |  | 1.126 |
| TRKB |  | 116.50 | 131.28 |  | 1.127 |
| JAK1 |  | 1190.50 | 1357.47 |  | 1.140 |
| PYK2 |  | 65.50 | 75.02 |  | 1.145 |
| EphA7 |  | 121.00 | 142.45 |  | 1.177 |
| EphA8 |  | 78.00 | 92.43 |  | 1.185 |
| MUSK |  | 78.00 | 92.88 |  | 1.191 |
| M-CSFR |  | 14.50 | 17.41 |  | 1.201 |
| JAK3 |  | 13.00 | 15.63 |  | 1.202 |
| HGFR |  | 58.00 | 70.11 |  | 1.209 |
| EphB2 |  | 52.00 | 62.96 |  | 1.211 |
| SCFR |  | 8.00 | 9.82 |  | 1.228 |
| SRMS |  | 229.50 | 283.55 |  | 1.236 |
| TXK |  | 225.50 | 289.36 |  | 1.283 |
| Hck |  | 325.00 | 417.96 |  | 1.286 |
| SYK |  | 50.50 | 68.77 |  | 1.362 |
| RET |  | 116.50 | 159.86 |  | 1.372 |
| ZAP70 |  | 73.50 | 118.78 |  | 1.616 |
| EphA2 |  | 16.50 | 26.79 |  | 1.624 |
