# Supplementary material for: CD73/NT5E is a target of miR-30a-5p and plays an important role in the pathogenesis of non-small cell lung cancer
Source: Mol Cancer. 2017 Feb 3;16:34. doi: 10.1186/s12943-017-0591-1 (PMC5291990; doi:10.1186/s12943-017-0591-1)
Supplement: Additional file 7: Table S3. — Differential expression of miRNAs and miR-30a-5p is downregulated in NSCLC. (DOC 57 kb) [file 12943_2017_591_MOESM7_ESM.doc]

**Supplementary Table 3**. Differential expression of miRNAs in NSCLC

|  | miRNA | Log2 Fold change^a^ | *p*-value | FDR^b^ | Chromosome |
| --- | --- | --- | --- | --- | --- |
| Upregulated | hsa-miR-3198 | 1.57 | 0.0002 | 0.013 | chr12 |
|  | hsa-miR-210 | 2.62 | 0.0045 | 0.045 | chr11 |
|  | hsa-miR-141-3p | 1.57 | 0.0050 | 0.048 | chr12 |
|  | hsa-miR-21-5p | 2.28 | 0.0040 | 0.045 | chr17 |
|  | hsa-miR-3676-5p | 1.66 | 0.0016 | 0.032 | chr17 |
|  | hsa-miR-96-5p | 2.69 | 0.0023 | 0.039 | chr7 |
|  | hsa-miR-3654 | 1.60 | 0.0011 | 0.030 | chr7 |
|  | hsa-miR-4286 | 2.36 | 0.0009 | 0.028 | chr8 |
|  | hsa-miR-200c-3p | 1.66 | 0.0019 | 0.035 | chr12 |
|  | hsa-miR-200b-3p | 1.83 | 0.0006 | 0.024 | chr1 |
|  | hsa-miR-183-5p | 3.60 | 0.0003 | 0.015 | chr7 |
|  | hsa-miR-429 | 1.71 | 0.0016 | 0.032 | chr1 |
|  | hsa-miR-146a-5p | 1.49 | 0.0048 | 0.047 | chr5 |
|  | hsa-miR-224-5p | 3.60 | 0.0029 | 0.043 | chrX |
|  | hsa-miR-193b-3p | 2.03 | 0.0039 | 0.045 | chr16 |
|  |  |  |  |  |  |
| Downregulated | hsa-miR-6075 | -1.30 | 0.0038 | 0.045 | chr5 |
|  | hsa-miR-4505 | -1.04 | 0.0032 | 0.045 | chr14 |
|  | hsa-miR-133b | -6.62 | 3.1×10^-5^ | 0.005 | chr6 |
|  | hsa-miR-451a | -3.28 | 0.0003 | 0.015 | chr17 |
|  | hsa-miR-4299 | -2.02 | 1.3×10^-4^ | 0.009 | chr11 |
|  | hsa-miR-30a-3p | -5.05 | 0.0016 | 0.032 | chr6 |
|  | hsa-miR-30a-5p | -2.85 | 6.0×10^-5^ | 0.006 | chr6 |
|  | hsa-miR-4728-5p | -1.09 | 0.0009 | 0.028 | chr17 |
|  | hsa-miR-335-5p | -3.80 | 0.0008 | 0.028 | chr7 |
|  | hsa-miR-575 | -1.49 | 0.0022 | 0.039 | chr4 |
|  | hsa-miR-126-3p | -2.09 | 0.0012 | 0.030 | chr9 |
|  | hsa-miR-5703 | -1.33 | 0.0034 | 0.045 | chr2 |
|  | hsa-miR-630 | -1.50 | 0.0026 | 0.041 | chr15 |
|  | hsa-miR-4532 | -1.17 | 0.0027 | 0.041 | chr20 |
|  | hsa-miR-4530 | -1.00 | 0.0038 | 0.045 | chr19 |
|  | hsa-miR-338-3p | -4.33 | 4.6×10^-6^ | 0.001 | chr17 |
|  | hsa-miR-4721 | -1.00 | 0.0014 | 0.032 | chr16 |
|  | hsa-miR-486-5p | -3.42 | 7.7×10^-5^ | 0.006 | chr8 |

^a^ After background subtraction and miRNAs normalization, we applied the Limma algorithm to filter the differentially expressed genes, and Log2FC was used to represent the ratio.

^b^ False discovery rate.
